# Using Auditory Steady State Responses to Outline the Functional Connectivity in the Tinnitus Brain

**DOI:** 10.1371/journal.pone.0003720

**Published:** 2008-11-13

**Authors:** Winfried Schlee, Nathan Weisz, Olivier Bertrand, Thomas Hartmann, Thomas Elbert

**Affiliations:** 1 Department of Psychology, University of Konstanz, Konstanz, Germany; 2 INSERM U821, Lyon, France; Victoria University of Wellington, New Zealand

## Abstract

**Background:**

Tinnitus is an auditory phantom perception that is most likely generated in the central nervous system. Most of the tinnitus research has concentrated on the auditory system. However, it was suggested recently that also non-auditory structures are involved in a global network that encodes subjective tinnitus. We tested this assumption using auditory steady state responses to entrain the tinnitus network and investigated long-range functional connectivity across various non-auditory brain regions.

**Methods and Findings:**

Using whole-head magnetoencephalography we investigated cortical connectivity by means of phase synchronization in tinnitus subjects and healthy controls. We found evidence for a deviating pattern of long-range functional connectivity in tinnitus that was strongly correlated with individual ratings of the tinnitus percept. Phase couplings between the anterior cingulum and the right frontal lobe and phase couplings between the anterior cingulum and the right parietal lobe showed significant *condition x group* interactions and were correlated with the individual tinnitus distress ratings only in the tinnitus condition and not in the control conditions.

**Conclusions:**

To the best of our knowledge this is the first study that demonstrates existence of a global tinnitus network of long-range cortical connections outside the central auditory system. This result extends the current knowledge of how tinnitus is generated in the brain. We propose that this global extend of the tinnitus network is crucial for the continuos perception of the tinnitus tone and a therapeutical intervention that is able to change this network should result in relief of tinnitus.

## Introduction

Chronic subjective tinnitus is described as an ongoing conscious perception of a sound in the absence of any physical sound source. About 5–15% of the population in western societies [1] report chronic tinnitus and in 1–3% the tinnitus affects their quality of life by disturbing sleep, impairing one's ability to concentrate at work, and affecting social interactions, as well as causing psychiatric distress [2]. Even though most of the tinnitus patients are able to localize their tinnitus to one or both ears, a transection of the auditory nerve does not eliminate the ongoing perception of the tinnitus sound [3], [4]. Thus it is hypothesized that the tinnitus sound is generated in the central nervous system and thus most of the tinnitus research of the last years concentrated on the central auditory system, especially on the cochlear nuclei, the inferior colliculus, the primary and the secondary auditory cortex [5]. However, there are also some lines of research that, using various methodologies, suggest an involvement of non-auditory structures: Mühlau and colleagues used Voxel-Based Morphometry (VBM) to study structural difference between tinnitus sufferers and controls and found a gray-matter changes within the auditory system (right posterior thalamus) and in non-auditory structures, namely the subcollosal region including the nucleus accumbens [6]. In another study using Positron Emission Tomography (PET), Mirz et al. compared the neuronal activity of tinnitus sufferers while they experienced their tinnitus with a condition where the tinnitus was suppressed by a masking sound or lidocaine application. In the tinnitus condition there was an increase of neuronal activity mainly in the right hemisphere, with a focus on middle frontal and middle temporal regions as well as in lateral in mesial posterior sites [7]. In a magnetoencephalographic (MEG) study, Weisz and colleagues found a reduction of alpha (8–12 Hz) power and an enhancement of delta (1.5–4 Hz) power in the resting state of tinnitus sufferers. These changes were more pronounced in the temporal regions but also significant for left frontal and right parietal areas [8]. These results suggest that the sensation of tinnitus is associated with neuronal activity in sensory auditory areas together with cortical regions subserving emotional, mnemonic and attentional functions. This has been hypothesized earlier by Jastreboff who stated [9] that sensory and emotional aspects of the tinnitus percept are integrated at higher levels of the nervous system and the prefrontal cortex has been suggested to be a potential candidate for it. Thus it seems reasonable to speak of a widespread tinnitus network that integrates the acoustic properties of the tinnitus sounds together with other aspects of the tinnitus such as attentional allocation, emotional evaluation and associated knowledge about the tinnitus as well as false beliefs about the potential danger of tinnitus. However, the concept of a network goes beyond the mere co-activation of these regions and implies functional connectivity between the nodes of the network. To the best of our knowledge, there is currently no study that showed this functional connectivity of a tinnitus network.

The goal of the current study was to probe the tinnitus network with an auditory stimulus that resembles the individual tinnitus tone of the subject and investigate the functional connectivity of the evoked network. We defined eight regions of interest, namely the left and right frontal lobe, the left and right temporal, the left and right parietal lobe, the anterior and the posterior cingulum and investigated the functional connectivity between those regions. To probe inter-regional coupling, we stimulated the subjects with 37 Hz amplitude-modulated (AM) tones and measured the distribution of the phase angle differences of the 37 Hz Steady-State Response between distinct brain regions. This measure (from zero to one) should increase the more the distribution within a unit circle deviates from uniformity, indicating phase coupling between the sources [10]. The great advantage of using AM tones is that the response frequency of interest is clearly defined in advance, as the AM of the sound evokes a brain response at exactly the same frequency [11]–[14].

In general, auditory stimulation does not only lead to a cortical response of the auditory system. For instance, it has been shown that the anterior temporal lobe and the inferior prefrontal cortex are activated during sound identification, whereas the inferior parietal cortex, the super parietal cortex and the frontal gyrus are activated during sound localization [15]–[18]. These brain responses to a sound can be evoked – at least partially – automatically and the involved regions might also be functionally connected. To separate this stimulus-evoked connectivities from the couplings that are specific for the tinnitus network, we used a design of three stimulation conditions (two control tones and a tinnitus tone) and compared the brain responses between a healthy control group and a tinnitus group. The carrier frequency of the tinnitus tone condition was matched to the individual pitch properties of the tinnitus sound while two control frequencies were chosen 1.1 and 2.2 octaves below. Carrier frequencies for the control group were simulated by randomized selection from a similar frequency range.

Consequently, we anticipated some sort of network response in all stimulation condition. However, in the tinnitus tone condition for the tinnitus group we expected to trigger the tinnitus network in addition to that (outlined in table 1).

**Table 1:** Illustration of the design and the expected responses.

	Control Tone 2	Control Tone 1	Tinnitus-tone
Tinnitus Group	stimulus-evoked network	stimulus-evoked network	stimulus-evoked network+Tinnitus Network
Control Group	stimulus-evoked network	stimulus-evoked network	stimulus-evoked network

We found a network that incorporates the right parietal cortex, the right frontal lobe and the anterior cingulum that was specific to the tinnitus condition. The strength of functional coupling between those regions correlated well with subjective ratings of tinnitus intrusiveness in the tinnitus-tone condition but not in the control conditions.

## Methods

### Subjects

Twelve individuals with chronic tinnitus (seven women; mean age±SD: 27.9±8.6, mean tinnitus duration in years±SD: 5.8±4.2) and 10 normal hearing controls (five women; mean age±SD 25.7±2.7) participated in the study. All participants were right-handed according to the Edinburgh Handedness Inventory (Oldfield, 1971). The study was approved by the institutional review board of the University of Konstanz, the participants were fully informed about the experimental procedure, and signed a written consent form prior to the experiment. After the experiment subjects were paid for their participation (15 €). All subjects were recruited at the University of Konstanz.

Subjective ratings of the tinnitus intrusiveness were assessed prior to the experiment with a widely used and neurophysiologically validated questionnaire [19], [20]. The tinnitus intrusiveness is one subscale of this questionnaire with a test-retest-reliability of .86. Detailed patient information are shown in table 2.

**Table 2:** Patient Information of the Tinnitus Sample.

Subject	Age	Sex	Tinnitus Intrusiveness	Aetiology	Tinnitus Duration	Tinnitus Side
1	29	M	NA	Unknown	1	Bilateral
2	38	F	5	Sudden hearing loss	14	Bilateral
3	32	F	3	Unknown	2	Right Ear
4	20	M	11	Unknown	2	Bilateral
5	24	M	7	Noise Trauma	3	Right Ear
6	22	F	2	Unknown	6	Bilateral
7	23	M	1	Noise Trauma	3	Bilateral
8	26	M	8	Borelia Infection	9	Bilateral
9	25	F	3	Unknown	6	Bilateral
10	50	F	7	Noise Trauma	12	Left Ear
11	23	F	2	Noise Trauma	4	Bilateral
12	23	F	10	Unknown	8	Bilateral

### Experimental Design and Apparatus

During the auditory stimulations the subjects watched stable pictures of neutral emotional content. This was done to focus their attention and keep them awake. The images were shown starting about one second before the tone started until about one second after the tone stopped. This was done to avoid the recording of visual-evoked potentials during the tone presentation. The same set of pictures was used in the control and the tinnitus group. The images were taken from the International Affective Picture System (IAPS). We selected pictures of neutral emotional content (low arousal, low valence) to avoid differential emotional responses. Both groups saw the same set of pictures. The inter-trial interval (ITI) varied between two to three seconds. During this pause the patients were encouraged to blink, so that they could avoid blinking during the stimulation. The procedure, including sending markers to the data acquisition system, was implemented in Psyscope [21] (http://psy.ck.sissa.it).

The steady-state signals were modulated with a modulation frequency of 37.1 Hz and a modulation depth of 100%. In the tinnitus group, the carrier frequency was matched to the individual pitch properties of the tinnitus sound while two control frequencies were chosen 1.1 and 2.2 octaves below. The difference of 1.1 and 2.2 octaves was chosen to avoid harmonics. Carrier frequencies for the control group were simulated by randomized selection from a similar frequency range. For removing clicks at on- and offset of the stimuli there was an on- and off-set ramp of 15 ms applied to the tones. The stimuli were presented with a sampling rate of 44'100 Hz. Each stimulus lasted 10 seconds and was randomly presented monaurally, 30 times per ear. The loudness of each tone was matched individually to a 1000 Hz AM-tone to ensure equal loudness perception in all conditions.

The auditory stimuli were generated outside the magnetically shielded room and conducted to the patient's ear via a flexible tubing sound delivery system with approximately linear filter properties. The visual stimuli were also generated outside the magnetically shielded room with a video beamer (DLA-G11E, JVC, Friedberg, Germany) and were projected onto a white projection field on the ceiling of the room using a mirror system.

### Audiometric Measures and Definition of the Tinnitus Frequency

The tinnitus sample underwent a series of audiometric tests to assess for hearing problems and the frequency spectrum of the tinnitus sound. These measures were used to define the “tinnitus tone” that was used for the experiment. All measures were done in a noise-reduced chamber prior to the experiment. Audiometric measures where carried out with a clinical audiometer (AC40 Clinical Audiometer, Audiometrics, Shreveport, LA) to determine the amount of hearing loss in the following frequencies: 250, 500, 1000, 1500, 2000, 3000, 4000, 5000, 6000, and 8000 Hz. For the diagnosis of dead regions on the cochlea, we conducted the Threshold Equalizing Noise (TEN) test developed by Moore et al. 2000 [22]. The idea of the TEN test is to measure damage of inner hair cells that cannot be diagnosed by normal clinical audiometry. Under normal conditions, hearing loss that is restricted to a small portion can be compensated by off-frequency listening. That is, hair cells of a neighboring undamaged region on the cochlea are activated by the sound. The TEN test accounts for this effect by presenting a threshold equalizing noise while audiometry is conducted. The same frequencies as in the clinical audiometry were tested. Two conditions must be fulfilled to speak of a dead region: The hearing threshold of the subject at a certain frequency must be at least 10 dB larger than the noise level and the threshold must be more than 10 dB above the normal hearing threshold.

To assess the individual tinnitus spectrum, we used an approach that was published by Norena and colleagues [23]. Pure tones of varying frequencies were presented to the subject one at a time. Again, the same frequencies as for the clinical audiometry were used here. Each trial consisted of two parts: In a first step the subject was requested to adjust the loudness of the tone such that it matched the perceived loudness of the tinnitus. In a second step the patient was asked to rate how much the tone belonged to the tinnitus percept on a scale between zero and ten. Overall each frequency was presented four times in a pseudorandom order. The first round, in which all 10 frequencies were presented once, was considered a practice round and was not considered in the analysis.

Normally, these spectrum ratings are not a single frequency, but rather a spectrum of frequencies. However, the experimental design that we used here needed a single carrier frequency for the “tinnitus tone” condition rather than an individual tinnitus spectrum to make it comparable to the other subjects and conditions. Also, the frequencies of the tinnitus spectrum typically overlap with the frequencies of substantial hearing loss.

However, in the ideal case, the tinnitus spectrum is characterized by a sharp increase for higher frequencies that finally reaches a plateau. As found earlier in our lab, the first frequency of this plateau is concordant with the front edge of the hearing loss region [24]. Thus, this frequency is reported to have strong similarity with the tinnitus perception and is mostly within normal hearing levels.

### Data Acquisition and Analysis

The data were recorded with a 148-channel whole-head magnetometer system (MAGNES 2500 WH, 4D Neuroimaging, San Diego, USA), installed in a magnetically shielded room (Vakuumschmelze Hanau, Germany). Artifact correction for heartbeats and eye blinks were performed using a semi-automated process implemented in BESA (MEGIS, Gräfelfing, Germany) prior to the following analysis. In this approach, the spatial topographies of relevant EOG and ECG activities are estimated in a first step. The resulting spatial vectors (estimated via PCA; normally one component for blinks and 2 components for ECG account for >90% of the topography) are added to the brain (forward) model. By this means the influence of the artifactual sources can be removed. The signals of each trial, recorded with a sampling rate of 678.17 Hz were averaged across artefact-free periods and projected to a source montage of eight regional neural sources using BESA. The source configuration was adjusted to the individual head size and consisted of temporal, orbitofrontal and parietal sources in both hemispheres, one source centered within the posterior cingulate cortex and one in the anterior cingulate cortex. After bandpass filtering (35 to 39 Hz), each trial was segmented in overlapping windows of 107.8 ms and averaged in the time domain to enhance the signal-noise-ratio.

The first 265 ms of each 10-second steady state response were discarded to avoid interference from early transient brain responses. The phase of the 37 Hz response was estimated with a fast Fourier transformation for each trial and source. The phase difference was calculated for all possible pairs of sources and phase synchrony was operationalized as mean length of the vector of the circular data (similar to Lachaux et al., 2000). After averaging and estimating the phase, a Fisher-z-transformation was applied to the individual phase-locking values. As a first step we calculated a mixed models ANOVA for every connection and the significant *group x condition* interaction effect guided us to the connections of interest for further analysis. Second, these connections were correlated with the individual ratings of the tinnitus intrusiveness. Two of these connections turned out to have significant correlations with the intrusiveness and in a third step they were entered into regression analysis explaining the tinnitus intrusiveness based on the observed phase synchronies.

## Results

In a first step we did a between-group comparisons of the tinnitus and the control group. Brain connectivities that discriminate between tinnitus and control tones were investigated without prior restrictions to specific regions. A second analysis within the tinnitus sample specifies those connectivities that reflect the variance in the subjective ratings of the tinnitus intrusiveness.

**Figure 1:**
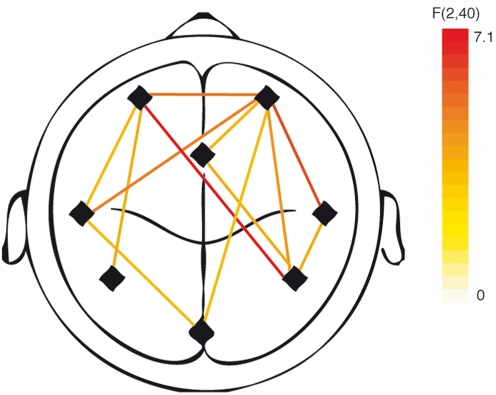
Long-range connectivities with a significant interaction effect *group x condition*. The data are presented in top view showing frontal, temporal and parietal sources in both hemispheres as well as one source at the anterior cingulate cortex and one posterior source. Line colours represent the strength of the interaction.

A mixed-models ANOVA (subject as random variable) was calculated with the factors *group* and *condition* for each inter-regional coupling. Significant interaction terms revealed a deviating synchronization pattern of the “tinnitus tone” and the control tones between the two groups (figure 1).

We found strong support for the assumption that individuals with tinnitus process the stimuli differently than controls. Abnormal connectivity was widely dispersed over the whole brain (figure 1). The right parietal source and frontal sites play a prominent role in this network of abnormal coupling. This complements previous evidence for an involvement of auditory and non-auditory regions in tinnitus patients [6]–[8], [25]. However, from this analysis we were unable to determine which of the effects are specific to the tinnitus tone and were not able to identify increases or decreases in synchronization. In order to map these interconnectivities, we correlated the strength of the phase synchronization with the subjective ratings of the tinnitus intrusiveness for all three stimulation conditions. Stimulation with the tinnitus-like tone is more likely to evoke the tinnitus network and synchronizations between the nodes of the network while control tones are less likely to evoke such a response.

**Figure 2:**
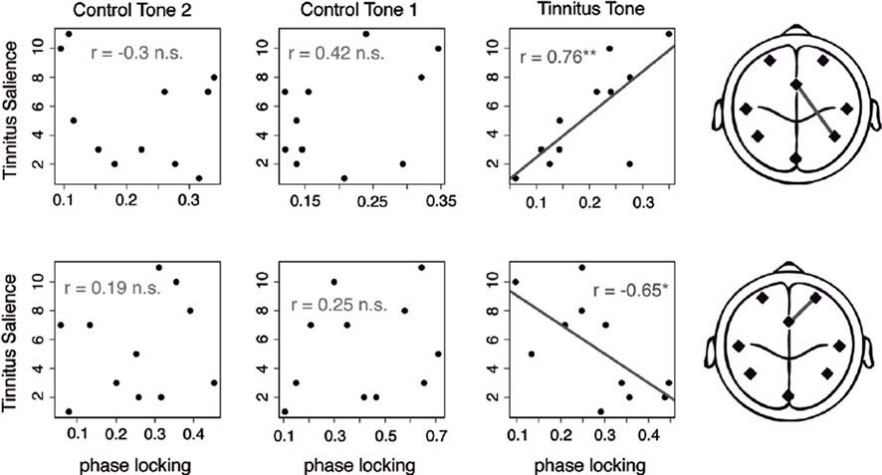
Inter-regional connectivities with an association between tinnitus intrusiveness and phase synchronization. The first row shows the scatterplots of the inter-regional connectivity between the right parietal and the anterior cingulate cortex across all stimulation conditions. Subjective ratings were positively correlated with the inter-regional phase synchronization when stimulated with the tinnitus tone. There was no correlation when the control tones were played. The second row depicts the same plots for the connectivity between right frontal and anterior cingulate cortex. The correlation between tinnitus intrusiveness and phase synchrony was negative. Again, there was no significant correlation between the two control conditions.

Two connections revealed a significant and also strong relationship between the strength of phase synchrony and tinnitus intrusiveness, both exclusively in the tinnitus tone condition applied to the left ear (see figure 2). The coupling between the right parietal source and the anterior cingulum was positively correlated with tinnitus intrusiveness (r = 0.75, p<0.001). The pair between the right frontal source and the anterior cingulum was negatively associated with tinnitus intrusiveness (r = −0.65, p = 0.03). Correlations between synchrony and intrusiveness at the control frequencies were all far from significance (p-values>0.2). This lends credibility to the assumption that the interconnectivities in figure 2 are part of a network related to tinnitus.

Theoretically, an oscillating single source could project its activity to two neighboring regions and thereby mimic synchronized activity between them. This possibility is unlikely for two reasons: a) We conducted the same statistics for the amplitude measures of the eight regional dipoles: Significant interactions of the same conditions (analogous to Figure 2) were only found for the left temporal and PCC source. Correlations with the tinnitus intrusiveness were not significant (all p>0.1). b) We tested the phase differences between the dipoles. If volume conduction is explanatory for the phase coupling, the phase values should be centered at 0° or 180°. This was not the case for either of the connectivities in figure 2, which is inconsistent with the volume conduction explanation. Phase differences of all tinnitus subjects are reported in the supporting information (figure S1 and S2).

Since there is a high percentage of tinnitus patients that also suffer from hearing loss, the study effects reported here might be confounded by damage to the hearing system. Because hearing loss triggers plastic changes in the tonotopic map of the auditory cortex, frequencies at the edge of the hearing loss region end up to be overrepresented [5]. Thus, a rational argument could be that the phase synchrony effects at the lesion edge are merely a result of the hearing loss rather than a function of the tinnitus saliency. To test this alternative we correlated the phase synchrony measures with various parameters of the audiometric tests. However, neither of these parameters seems to be associated with the phase connectivities nor with the individual tinnitus intrusiveness rating. a) The hearing threshold (dB SPL) at the carrier frequency of the sound in the respective condition was not associated with neither of the phase synchronies nor with the tinnitus intrusiveness ratings (all p>0.2). b) The overall amount of hearing loss (dB SPL), as well as the maximal hearing loss, were neither associated with the phase synchronies nor with the tinnitus intrusiveness ratings (all p>0.5). c) The maximal steepness of the hearing loss function (Δ dB SPL/octave) was neither associated with the phase synchronies nor with the tinnitus intrusiveness ratings (all p>0.3).

Since stimulation parameters varied between subjects, the reported effects could also result from variations in the carrier frequency of the stimuli (e.g. higher carrier frequencies appear to be more salient). To test this possibility we correlated the carrier frequencies with the phase lockings of the ACC-Right Frontal and the ACC-Right Parietal connection but found no significant correlation (all p>.2). However, it has to be noted that this tests cannot completely rule out the possibility that effects of hearing loss confounded these results. This is due to potential recruitment effects, which are a by-product of sensorineural hearing loss.

Taken these results together, we found a result pattern that includes long-range synchronization (connection ACC – Right Parietal Area) with long-rang desynchronization (connection ACC – Right Frontal Area) that very strongly correlates with tinnitus distress while major confounds can be empirically excluded. To integrate these two different qualities of connectivity into one model, we calculated a regression analysis taking the interconnectivities as independent and the tinnitus intrusiveness as a dependent variable. This model demonstrated a near perfect fit to the data with an adjusted R-square of 0.82 (F(2,8) = 24.37, p<.001), while there was no indication of a correlation between the regressors (p>0.6), suggesting independence between these components.

## Discussion

To the best of our knowledge this is the first study that demonstrates long-range functional connectivity in tinnitus. The phase coupling between the anterior cingulum and the right frontal lobe and the phase coupling between the anterior cingulum and the right parietal lobe showed significant condition x group interactions and showed meaningful correlations with the subjective ratings of the individual tinnitus distress. These correlations were only found in the tinnitus condition and not in the control conditions, which lends confidence that, these couplings are related to a global network that is involved in the processing of the tinnitus percept. The phase synchronization between ACC and right frontal was inversely correlated with tinnitus intrusiveness while the phase synchronization between ACC and right parietal was positively correlated with tinnitus intrusiveness.

The source montage that we used in this study covers main areas of interest in the cortex, however it does not allow an interpretation of the precise location of the coupled sources. This is also because of technical constraints that are inherent to the inverse modeling used in MEG. For instance we cannot decide which part of the prefrontal cortex is responsible for the decoupling with ACC area. Thus we can only roughly interpret the functional meaning of this network. However, the regions that we found to be coupled in our study have been frequently found to be involved and co-activated in studies on stimulus salience [26]–[29] and it has been shown in these studies that the salience network is biased to the right hemisphere. In a fMRI study using visual, auditory and tactile stimuli Downar and colleagues [26] identified a frontal-parietal-cingulate network that may serve to identify and evaluate salient stimuli and this network seems to be independent of the sensory modality. They tested this network again [27] using painful and non-painful stimuli and found a sustained activation of the frontal-parietal-cingulate network during painful stimuli and only transient response of these regions at on- and offset of the non-painful stimuli. Mevorach and colleagues [29] tried to interrupt this network and investigated the attention towards salient stimuli during repetative Transcranial Magnetic Stimulation (rTMS) of the posterior parietal cortex (PPC). Stimulation to the right PPC disturbed the attentional mechanisms towards the salient stimulus while stimulation to the left PPC had an impact on moving the attention away from the salient stimulus. Mesulam [28] suggested that spatial attention–independent of the modality–is processed in a large-scale distributed network that consisted at the cortical level of the cingulate gyrus, the posterior parietal cortex and the frontal eye field. The idea of a widely distributed cortical network is also described in the global neuronal workspace model by Dehaene [30]. They postulate the existence of cortical workspace neurons that are distributed over distant areas of the cortex and connected via long-range excitatory axons. Information that is processed within this network can be easily accessed by various brain systems and it is hypothesized that this workspace is the basis for conscious perception. According to this model, conscious perception of a sensory stimuli needs 1) activation of the respective sensory system and 2) and entry into the global workspace. Global workspace neurons are thought to be localized in all sensory areas and additionally in the prefrontal, parietal and cingulate cortices. Cytoarchitectonic studies support the idea that workspace neurons are localized in these areas: Long-distance cortico-cortical connections originate mainly from pyramidal cells in the layers II and III and that these layers are thicker in prefrontal and parietal cortices. Furthermore it has been shown in the monkey that these regions are strongly interconnected and entertain also connections to the anterior and posterior cingulum as well as to the temporal region, the hippocampus and subcortical regions [31]. Following the logic of the global workspace model one could expect also significant activation of the connections between the temporal cortex and the global workspace brain regions. Accordingly, since all subjects were stimulated with tones well above the perception threshold we didn't expect a divergent activation of these connectivities in our experiment. Hence, all subjects had a conscious perception of the tones in all conditions, however the activation within the prefrontal-parietal-cingulate network differentiated between groups and conditions.

Salient sensory stimuli preferentially draw our attention and enter our consciousness. Thus it is not surprising that studies that investigate stimulus salience, attention or consciousness all come up with similar models. They all suggest a widespread cortical network that integrates regions as distant as the frontal, parietal, and cingulated cortex and many of them suggest a tendency to the right hemisphere. In this study we found phase synchronizations between ACC, the right frontal and the right parietal to be strongly correlated with tinnitus intrusiveness. Tinnitus intrusiveness is defined by how bothersome and obtrusive the tinnitus is perceived, the potency of the tinnitus sound to automatically draw attentional resources, and the potential of affecting everyday behavior. Thus, it is likely that the network that we observed here represents a more general salience network that is activated by the individually perceived saliency of the tinnitus sound. Further studies will be needed to disentangle – if possible – the salience network from a specific tinnitus network.

However, it is still puzzling why the coupling between ACC and the right frontal lobe correlates negatively with tinnitus intrusiveness. This might be related to the fact that the ACC has been associated with emotional evaluation, for instance in studies on pain [32]–[36] while the frontal regions that are connected to the ACC are involved in controlling and correction of behavior [37]–[40]. Thus, a disconnection of the “control center” from the affective component could enhance the intrusiveness of the tinnitus tone. This, however remains speculation.

Most of our recruited tinnitus subjects reported bilateral tinnitus and there were only 3 subjects with unilateral tinnitus (two subjects reported tinnitus at the right ear, one subject at the left ear). Thus, we were not able to statistically differentiate between bilateral and unilateral tinnitus, neither between left-sided and right-sided tinnitus. Interestingly, even though most of the patients reported bilateral tinnitus, the correlations between tinnitus intrusiveness and phase-synchrony were lateralized to the right hemisphere rather than being symmetric. We suspect that this is an effect of hemispheric specialization of the distress network that is independent from the tinnitus side. Indeed it has been shown in other studies that the cortical networks involved in detecting salient stimuli are lateralized to the right hemisphere regardless of the location of the stimulation [26], [28].

Altogether the results suggest the existence of a global tinnitus network of long-range cortical connections outside the central auditory system. We hypothesize that this global network is crucial part in understanding the tinnitus. Let's assume that a tinnitus sound would be generated in the auditory system but does not engage in long-range couplings across the cortex. Using the words of the Dehaene-model, the tinnitus would not enter into the global workspace and thus the tinnitus would stay preconscious [41]. Thus, the patient would not be aware of the tinnitus sound, the tinnitus could not affect the daily life and would not cause any psychological problems. Thus, we propose that a therapeutical intervention that is able to change the global extend of the tinnitus network should result in relief of tinnitus awareness.

To reach this goal, we need further studies that scrutinize the architecture of the tinnitus with a technique that allows more precise localization of the nodes of the network. It might be that this network architecture changes with duration of tinnitus. If this is the case it would be important to know the nature of this change to design treatment strategies that specifically target this network. In order to challenge our assumption with an experimental manipulation, a short-term suppression of the tinnitus tone could be applied with residual inhibition, rTMS or lidocaine administration and this should change the activation pattern of the global tinnitus network. Also, if there is a tinnitus network that encodes the awareness of the phantom tinnitus sound, this network should also be active in the resting state.

## Supporting Information

Figure S1 Degrees of the phase differences for the Right Frontal-ACC connectivities. There is a rose plot of 30 trials for each tinnitus subjects. Rose plots are sorted according to the tinnitus intrusiveness.
(0.22 MB PDF)

Figure S2 Degrees of the phase differences for the Right Parietal-ACC connectivities. There is a rose plot of the directionalities over 30 trials for each tinnitus subjects. Rose plots are sorted according to the tinnitus intrusiveness.
(0.22 MB PDF)
