# Correction: Using Auditory Steady State Responses to Outline the Functional Connectivity in the Tinnitus Brain

**DOI:** 10.1371/annotation/c71826c3-da07-42e8-a563-bde0257a0845

**Published:** 2008-12-03

**Authors:** Winfried Schlee, Nathan Weisz, Olivier Bertrand, Thomas Hartmann, Thomas Elbert

The funding information for this paper was omitted. The complete funding information should say: This work was supported by the Deutsche Forschungsgemeinschaft (DFG) and the Tinnitus Research Initiative (TRI). The funders had no role in study design, data collection and analysis, decision to publish, or preparation of the manuscript.

