# Supplementary material for: Using Auditory Steady State Responses to Outline the Functional Connectivity in the Tinnitus Brain
Source: PLoS One. 2008 Nov 13;3(11):e3720. doi: 10.1371/journal.pone.0003720 (PMC2579484; doi:10.1371/journal.pone.0003720)
Supplement: Figure S1 — Degrees of the phase differences for the Right Frontal-ACC connectivities. There is a rose plot of 30 trials for each tinnitus subjects. Rose plots are sorted according to the tinnitus intrusiveness. (0.22 MB PDF) [file pone.0003720.s001.pdf]

Right Frontal - ACC

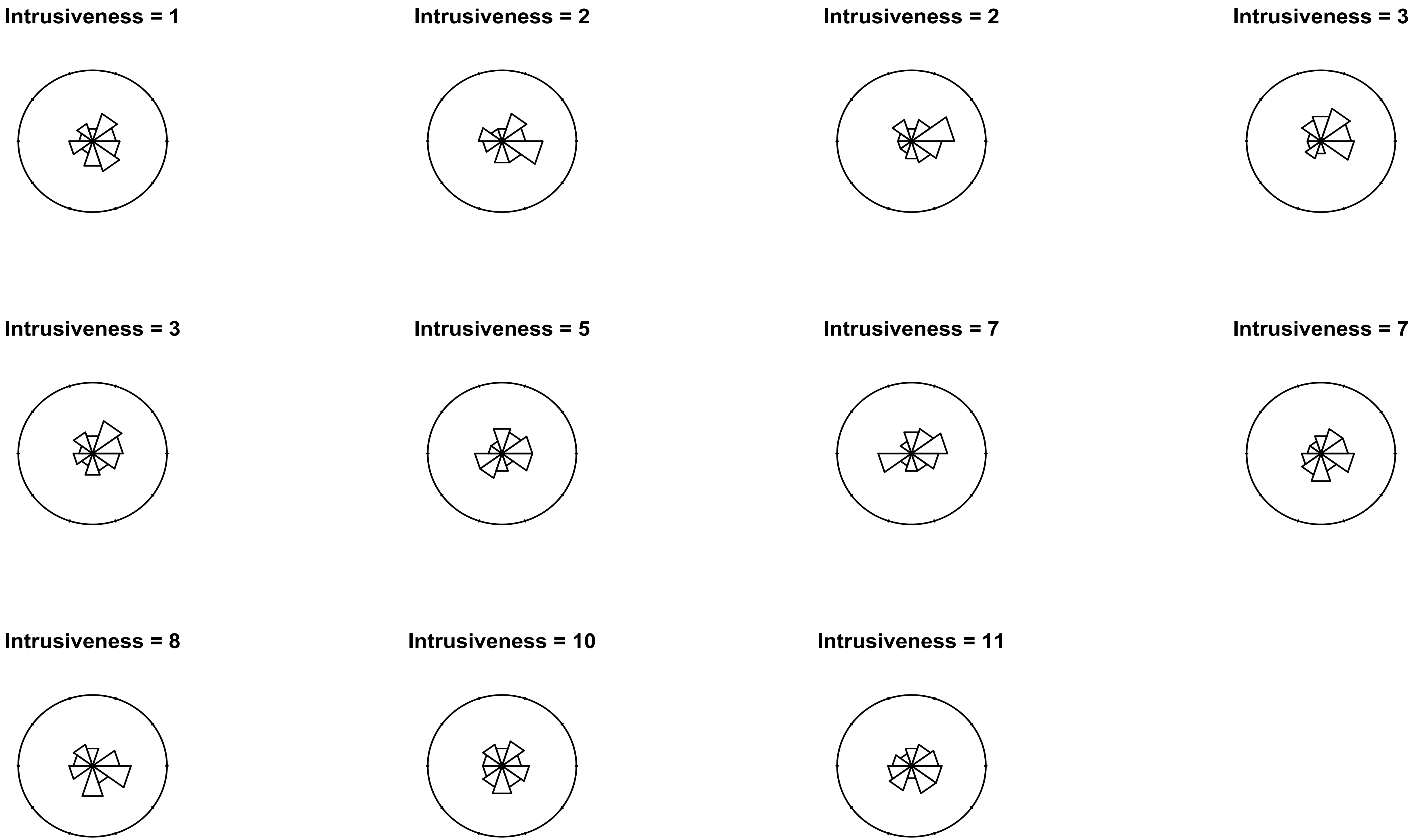

Figure S1.
Degrees of the phase differences for the Right Frontal-ACC connectivities. There is a rose plot of the directionalities over 30 trials for each tinnitus subjects. Rose plots are sorted according to the tinnitus intrusiveness.
